# GCH1 (rs841) polymorphism in the nitric oxide-forming pathway has protective effects on obstructive sleep apnea

**DOI:** 10.1038/s41598-019-55244-1

**Published:** 2019-12-09

**Authors:** Samaneh Sheikhi Kouhsar, Mohammadreza Bigdeli, Yadollah Shakiba, Khosro Sadeghniiat

**Affiliations:** 1 Department of Animal Sciences and Biotechnology, Faculty of Life Sciences and Biotechnology, Shahid Beheshti University, Tehran, Iran; 2 Institute for Cognitive and Brain Sciences, Shahid Beheshti University, Tehran, Iran; 3 Regenerative Medicine Research Center, Kermanshah University of Medical Sciences, Kermanshah, Iran; 4 Occupational Sleep Research Center, Baharloo Hospital, Tehran, Iran

**Keywords:** Genetic predisposition to disease, Genetics research

## Abstract

Several studies have recently investigated the contribution of genetic factors in obstructive sleep apnea (OSA). Patients with OSA suffer from a reduction in nitric oxide (NO) serum level. This study investigated rs841, A930G p22phox, and rs1799983 polymorphisms in three critical genes involved in NO formation. A total of 94 patients with OSA and 100 healthy controls were enrolled into the study. Results showed there was no association between rs841, A930G p22phox and rs1799983 polymorphism and the risk of OSA (*P* = 0.51, *P* = 0.4 and *P* = 0.33, respectively). Moreover, rs841 GA genotype had a reverse relationship with the severity of OSA (*P* = 0.005). On the other hand, rs841 GA and A930G p22phox AA genotypes had a protective effect on daytime sleepiness in OSA patients (*P* = 0.01and *P* = 0.02, respectively). Additionally, the combination of rs841 and A930G p22phox (AG/AG and AG/AA) genotypes was significantly associated with a reduction in daytime sleepiness in OSA patients (*P* = 0.03 and *P* = 0.03, respectively). According to the results of our study, GA genotype of rs841 and GA/AA genotypes of A930G p22phox polymorphisms significantly reduced the severity of the problem and daytime sleepiness in OSA patients.

## Introduction

Obstructive sleep apnea (OSA) is a common sleep disorder^1,2^, which is characterized by repetitive pharyngeal obstruction, leading to apnea and hypopnea during sleep^3^. Headache, Fatigue, excessive daytime sleepiness, non-refreshing sleep, irritability, and decreased cognitive functions are the common symptoms of OSA^4,5^. The prevalence of undiagnosed OSA among the general population is estimated to be 5%. In addition, the prevalence of undiagnosed moderate to severe OSA among a sample of general population in Western Australia was 9%. Nonetheless, it is estimated that only 40% of people with OSA are diagnosed^6,7^. Untreated OSA is associated with different health complications, including metabolic disorder^8^, cognitive impairment^9^, depression^10^, and cardiovascular diseases^11^; this disorder also has an economic burden on community^12^. OSA is a multifactorial disorder and several genetic studies have provided evidence for the possible association between OSA and genetic factors^13,14^.

Nitric Oxide (NO) is synthesized from L-Arginine substrate by a family of nitric oxide synthase (NOS) enzymes. In this process, NADPH and O_2_ serve as co-substrates and 6-tetrahydrobiopterin (BH4) acts as a co-factor^15^. NO is a signaling molecule in the human body that is involved in many physiological and pathological processes^16,17^. NO plays an important role in neural signaling, immune response, vasodilation, and modulating insulin sensitivity^18^. NO deficiency is involved in the pathogenesis of multiple diseases such as hypertension^19^, diabetes mellitus^20^, stroke^21^, and OSA^22^. Nitric Oxide derivatives (serum nitrites and nitrates**)** and L-Arginine plasma levels decrease in patients with OSA, however, they increase after continuous positive airway pressure (CPAP) therapy^23,24^. Chronic sleep deprivation and repetitive hypoxia / reoxygenation in patients with OSA impairs endothelial function through reducing NO bioavailability and increasing oxidative stress and inflammation^11^. Therefore, changes in substrate, enzymes, and co-factors that are involved in NO formation may decrease NO levels in patients with OSA. Several functional polymorphisms have been identified in NO-forming pathway^25,26^. eNOS is encoded by NO3 gene, and some polymorphisms have been reported for NOS3 gene, including rs1799983, intron 4a/b, rs2070744, etc. They play a role in different diseases such as OSA^27,28^. According to previous studies, G894 T (rs1799983) variant is responsible for NO reduction^29^.

GTP cyclohydrolase 1 (GCH1) catalyzes the biosynthesis of BH4 that is an essential cofactor in the synthesis of NO. Moreover, rs841 polymorphism of GCH1 is involved in neuropathic pain, attention, and stroke^30–32^. So far, no study has investigated the association between this polymorphism and OSA. NADPH oxidase is another factor which is involved in NO formation and is identified as the major source of reactive oxygen species (ROS). It is a multicomponent enzyme consisting of catalytic subunits and cytosolic proteins. Among catalytic subunits, p22phox is a physical conduct for transferring electrons across the membrane and is critical for the enzymatic activity. On the other hand, p22phox subunit polymorphism is identified as a factor involved in OSA and cardiovascular diseases^33,34^.

This study investigated the association between OSA and rs841(G > A) in GTP cyclohydrolase I (GCH1), A930G p22phox (G > A) in NADPH Oxidase, and rs1799983 (G > T) in eNOS polymorphisms.

To the best of our knowledge, this study is the first research investigating the association between GCH1 polymorphism and OSA, as well as the relationship between rs841, A930G p22phox, and 1799983 in Iranian people.

## Results

### Genotypes, allele frequencies, and risk of OSA

Table 1 presents the data collected on patients’ and controls’ age, gender, BMI, and the data collected via STOP-BANG and Epworth Sleep Scale questionnaires. The collected data were used to assess the association between three polymorphisms in three different genes and the risk of OSA. All genotypes observed in cases and controls were consistent with Hardy–Weinberg equilibrium (HWE) (*P* > 0.05). Table 2 presents the genotype distributions and allele frequencies of rs841(GCH1), A930G p22phox (NADPH Oxidase), and rs1799983 (eNOS) polymorphisms. Based on the results, genotypes and allele frequencies of rs841 (G > A), A930G p22phox (G > A) and rs1799983 (G > T) polymorphisms had no significant association with the risk of OSA (A *vs*. G: OR = 0.74, 95% CI = 0.3–1.81; *P* = 0.51, A *vs*. G: OR = 0.82, 95% CI = 0.53–1.28; *P* = 0.4 and T *vs*. G: OR = 2.12, 95% CI = 0.46–9.74; *P* = 0.33, respectively).

**Table 1:** Characteristics of patient and control groups.

Characteristics	Control N = 100	Patient N = 94	Pvalue
Men n (%)	79 (79%)	75 (80%)	0.89
Age	42.74 ± 7.76	44.3 ± 11.45	0.26
BMI	26.77 ± 3.9	29.14 ± 4.5	0.000
STOP-BANG	1.2 ± 0.55	3.85 ± 1.45	0.000
ESS	1.3 ± 1.56	9.42 ± 6.00	0.000

BMI: Body Mass Index, ESS: Epworth Sleep Scale. Characteristics are defined by Mean ± Standard Deviation.

**Table 2:** Genotype distribution and allele frequency in OSA and controls.

SNP	Genotype/Allele	Control	OSA	Crude OR	P value	Adjusted OR	P value
		N = 100	N = 94	(95% CI)		(95% CI)	
Rs841	GG	63 (63%)	59 (62.8%)	1.00 (Reference)			
	GA	33 (33%)	32 (34%)	1.03 (0.55–1.92)	0.9	1.64 (0.3–8.79)	0.56
	AA	4 (4%)	3 (3.2%)	0.8 (0.19–3.09)	0.77	1.59 (0.28–8.83)	0.59
	Dominant						
	GG	63 (63%)	59 (62.8%)	1.00 (Reference)			
	GA + AA	37 (37%)	35 (37.2%)	1.01 (0.56–1.79)	0.97	0.93 (0.5–1.71)	0.82
	Recessive						
	GG + GA	96 (96%)	91 (96.8%)	1.00 (Reference)			
	AA	4 (4%)	3 (3.2%)	0.79 (0.19–3.01)	0.76	0.61 (0.11–3.25)	0.56
	G	159 (79.5%)	150 (79.8%)	1.00 (Reference)			
	A	41 (20.5%)	38 (20.2%)	0.98 (0.6–1.58)	0.94	0.74 (0.3–1.81)	0.51
A930G p22phox	GG	29 (29%)	27 (28.7%)	1.00 (Reference)			
	GA	48 (48%)	53 (56.4%)	1.18 (0.6–2.33)	0.6	1.18 (0.6–2.32)	0.63
	AA	23 (23%)	14 (14.9%)	0.65 (0.26–1.43)	0.32	0.68 (0.28–1.68)	0.41
	Dominant						
	GG	29 (29%)	27 (28.7%)	1.00 (Reference)			
	GA + AA	71 (71%)	67 (81.3%)	1.01 (0.55–1.86)	0.96	1.03 (0.54–1.97)	0.92
	Recessive						
	GG + GA	77 (77%)	80 (85.1%)	1.00 (Reference)			
	AA	23 (23%)	14 (14.9%)	0.58 (0.28–1.23)	0.15	0.61 (0.28–1.34)	0.22
	G	106 (53%)	107 (57%)	1.00 (Reference)			
	A	94 (47%)	81 (43%)	0.85 (0.57–1.26)	0.43	0.82 (0.53–1.28)	0.4
Rs1799983	GG	69 (69%)	51 (54.2%)	1.00 (Reference)			
	GT	28 (28%)	37 (39.4%)	1.78 (0.96–3.24)	0.06	1.55 (0.82–2.93)	0.17
	TT	3 (3%)	6 (6.4%)	2.7 (0.71–10.17)	0.15	3.57 (0.82–15.48)	0.08
	Dominant						
	GG	69 (69%)	51 (54.2%)	1.00 (Reference)			
	GT+TT	31 (31%)	45 (45.8%)	1.96 (1.11–3.55)	0.02	3.17 (0.07–1.14)	0.93
	Recessive						
	GG + GT	97 (97%)	88 (83.6%)	1.00 (Reference)			
	TT	3 (3%)	6 (6.4%)	2.2 (0.57–8.2)	0.26	0.32 (0.07–1.37)	0.12
	G	166 (83%)	139 (74%)	1.00 (Reference)			
	T	34 (17%)	49 (26%)	1.72 (1.04–2.81)	0.02	2.12 (0.46–9.74)	0.33

SNP: Single Nucleotide Polymorphism, OSA: Obstructive Sleep Apnea, OR: Odd Ratio, CI: Confidence Interval. Adjusted odds ratio were adjusted for body mass index.

### Genotypes and severity of OSA

In order to conduct further assessment, we divided OSA patients into two groups. There were 43 patients in severe group and 51 patients in mild-to-moderate group. Table 1 in appendix presents the polysomnographic parameters in patients. Statistical analysis did not show a significant difference between the severe and non-severe OSA patients in terms of the genotypes distribution of A930G p22phox (NADPH Oxidase) and rs1799983 (eNOS) polymorphism (*P* > 0.05, Table 3). Interestingly, as shown in Table 3, for the first time we found a significant difference between the severe and mild-to-moderate OSA patients in terms of the genotype of rs841 (GCH1), where GA genotype was more frequently observed in the mild-to-moderate OSA patients (Crude OR = 0.3, 95% CI = 0.12–0.78; *P* = 0.01, after adjusting for age, gender, BMI, OR = 0.21, 95% CI = 0.07–0.62; *P* = 0.005). The results showed that GA genotype of rs841 (GCH1) reduced the severity of OSA in patients; moreover, this genotype of rs841 had a protective effect in patients with OSA.

**Table 3:** Association between rs841, A930G p22phox, rs1799983 genotypes and the severity of OSA.

SNP	Genotype	Non-severe	Severe	Crude OR	P value	Adjusted OR	P value
		N = 51	N = 43			(95% CI)	
Rs841	GG	26	33	1.00 (Reference)			
	GA	23	9	0.3 (0.12–0.78)	0.01	0.21 (0.07–0.62)	0.005
	AA	2	1	0.3 (0.02–3.58)	0.44	0.05 (0.002–1.32)	0.07
A930G p22phox	GG	14	13	1.00 (Reference)			
	GA	31	22	0.76 (0.3–1.92)	0.57	0.61 (0.22–1.7)	0.35
	AA	6	8	1.43 (0.38–4.7)	0.58	1.14 (0.27–4.84)	0.85
Rs1799983	GG	28	23	1.00 (Reference)			
	GT	18	19	1.28 (0.55–3.03)	0.56	0.92 (0.36–2.31)	0.86
	TT	5	1	0.24 (0.01–2.11)	0.18	0.33 (0.33–3.36)	0.35

SNP: Single Nucleotide Polymorphism, OSA: Obstructive Sleep Apnea, OR: Odd Ratio, CI: Confidence Interval. Adjusted odds ratio were adjusted for age, gender and body mass index.

### Genotypes and daytime sleepiness in OSA

We investigated the association between the three genetic polymorphisms involved in NO formation and daytime sleepiness in OSA patients. We divided patients into two groups, patients with daytime sleepiness (n = 72) and patients without daytime sleepiness (n = 22) (Table 4). Assessing rs841 (GCH1), the frequency of GA genotype was significantly higher in patients without daytime sleepiness, as compared with patients with daytime sleepiness (Crude OR = 0.27, 95% CI = 0.1–0. 8; *P* = 0.01, after adjustment for age, gender, BMI OR = 0.23, 95% CI = 0.07–0.7; *P* = 0.01). Furthermore, assessing A930G p22phox, there was a significant difference between the patients without daytime sleepiness and the patients with daytime sleepiness in terms of genotype distribution; according to the results, AA genotype decreased daytime sleepiness in patients and had a protective effect (Crude OR = 0.23, 95% CI = 0.06–0.95; *P* = 0.04, after adjustment for age, gender, BMI OR = 0.14, 95% CI = 0.02–0.8; *P* = 0.02). There was no association between rs179983 (eNOS) genotypes and daytime sleepiness in the two groups of patients (*P* > 0.05).

**Table 4:** Association between rs841, A930G p22phox, rs1799983 genotype and daytime sleepiness in OSA.

SNP	Genotype	Non-sleepy	Sleepy	Crude OR	P value	Adjusted OR	P value
		N = 22	N = 72	(95% CI)		(95% CI)	
Rs841	GG	9 (40.9%)	51 (70.8%)	1.00 (Reference)			
	GA	12 (54.5%)	19 (26.4%)	0.27 (0.1–0.8)	0.01	0.23 (0.07–0.7)	0.01
	AA	1 (4.5%)	2 (2.8%)	0.35 (0.03–5.64)	0.39	0.3 (0.01–5.08)	0.4
A930G p22phox	GG	4 (18.2%)	23 (31.9%)	1.00 (Reference)			
	GA	12 (54.5%)	41 (56.9%)	0.59 (0.19–1.99)	0.4	0.56 (0.15–2.07)	0.38
	AA	6 (27.3%)	8 (11.2%)	0.23 (0.06–0.95)	0.04	0.14 (0.02–0.8)	0.02
Rs1799983	GG	14 (63.63%)	37 (51.4%)	1.00 (Reference)			
	GT	6 (27.27%)	31 (43%)	1.95 (0.66–5.81)	0.21	1.79 (0.58–5.52)	0.3
	TT	2 (9%)	4 (5.6%)	0.75 (0.16–4.34)	0.76	1.61 (0.23–11.03)	0.62

SNP: Single Nucleotide Polymorphism, OSA: Obstructive Sleep Apnea, OR: Odd Ratio, CI: Confidence Interval. Adjusted odds ratio were adjusted for age, gender and body mass index.

### Association between genotype combinations and daytime sleepiness in OSA patients

Interactions between polymorphisms within genes involved in the reduction of daytime sleepiness in OSA patients were investigated using the logistic regression analysis and the results showed a significant relationship between rs841 and A930G p22phox in two genotypes combination (Crude OR = 0.16, 95% CI = 0.02–0.98; *P* = 0.04 and Crude OR = 0.09, 95% CI = 0.009–0.97; *P* = 0.04, after adjustment for age, gender, BMI OR = 0.11, 95% CI = 0.01–0. 8; *P* = 0.03 and OR = 0.05, 95% CI = 0.003–0.83; *P* = 0.03, respectively). The combinations of rs841 GA genotype and A930G p22phox GA/AA genotype were significantly associated with a reduction in daytime sleepiness in patients with OSA, as compared with the reference combination of rs841 GG and A930G p22phox GG genotype (Table 5). The combinations of other genotypes did not result in a significant difference (*P* > 0.05).

**Table 5:** Distribution of combined genotypes in sleep and non-sleep OSA.

Genotype		Non-sleepy	Sleepy	Crude OR (95% CI)	P value	Adjusted OR (95% CI)	P value
rs841	A930G						
GA	GA	7	8	0.16 (0.02–0.98)	0.04	0.11 (0.01–0.8)	0.03
GA	AA	3	2	0.09 (0.009–0.97)	0.04	0.05 (0.003–0.83)	0.03

OR: Odd Ratio, CI: Confidence Interval. Adjusted odds ratio were adjusted for age, gender and body mass index.

## Discussion

Over the past two decades, public awareness about the importance of sleep and its related disorders has increased significantly^35^. In this work, we investigated the association between the susceptibility to OSA and GCH1 (rs841), NADPH oxidase (A930G p22phox (CYBA)) and endothelial NOS (rs1799983) polymorphisms. These genes play a role in nitric oxide formation^36^. To our knowledge, this was the first study that assessed the association between rs841 (GCH1) polymorphism and the risk of OSA. Some studies have shown that rs841 polymorphism is associated with the risk of ischemic stroke, endothelial dysfunction, and oxidative stress in patients with type 2 diabetes mellitus^32,37^. Interestingly, our results indicated no association between rs841 and the susceptibility to OSA; on the contrary, GA genotype of this polymorphism reduced the severity of the disease and daytime sleepiness in patients with OSA. Moreover, we did not find any relationship between A930G p22phox polymorphism and the risk of OSA; however, according to the results of a study by Pierola *et al*., this polymorphism plays an important role in genetic susceptibility to OSA^33^. In contrast, AA genotype of A930G p22phox polymorphism prevented daytime sleepiness in patients with OSA. The analysis of data collected in our study showed that T allele of rs1799983 polymorphism was not associated with increased risk of OSA. Bayazit *et. al*.’s study showed that eNOS4 polymorphism was not associated with OSA, while eNOS296 polymorphism was associated with OSA susceptibility. In this study, there was no relationship between eNOS4, eNOS296 polymorphisms and polysomnography parameters, diabetes mellitus, coronary artery disease, arrhythmia, hypertension, hypercholesterolemia, and smoking^28^. NO reduces in OSA patients, treatment with CPAP ameliorate endothelial nitric oxide release and vasodilation^38^.

Several studies have indicated a reduction in nitric oxide bioavailability in OSA patients^3,39,40^. BH4 is an essential co-factor required for the activation of all the three nitric oxide synthases; changes in this co-factor can affect NO formation^41^. GTP Cyclohydrolas 1 (GCH1) is a rate-limiting enzyme in the BH4 synthesis^42^. Therefore, changes in GCH1 gene could decrease or increase BH4 availability for NOS. Some studies have demonstrated that GCH1 rs841 polymorphism has a similar effect on BH4 levels in plasma and vascular tissues, and acts as a pain-protective haplotype of GCH1^43^. This polymorphism reduces BH4 levels in people with cardiovascular diseases, results in a reduction in NO, and increase superoxide production^42^. Given the protective effect of this polymorphism and other haplotypes of GCH1, it could be concluded that rs841 moderately reduces GCH1 expression and BH4 production^44^. Cycles of intermittent hypoxia, as a sign of OSA, promote oxidative stress and enhance the production of reactive oxygen species^39^. NADPH oxidase is a membrane-bound complex enzyme with cytosolic subunits (Rac, p47phox, p67phox) that are linked to catalytic membrane subunits (Nox, p22phox) to facilitate the superoxide production^45^. P22phox subunit plays an important role in the normal function of enzymes^46^. Recent studies have demonstrated that several polymorphisms of p22phox gene (CYBA) are associated with increased oxidative stress and cardiovascular diseases^32,47,48^. According to Pierola *et al*., patients with GA and GG genotypes of A930G p22phox polymorphism are more at risk of OSA. A930G p22phox polymorphism changes the expression of p22phox, in addition G allele increases p22phox expression and oxidative stress. A-930G polymorphism is associated with sleep apnea independently of sympathetic activation, obesity, hypertension, hyperlipidemia and diabetes mellitus^33^. A meta-analysis study indicated that A930G polymorphism might be a protective factor for hypertension^49^. Based on another study, NO production is lower in hypertensive patients with GG genotype of A930G polymorphism^46^, that could indicate that patients with GA/AA genotype produce more NO than patients with GG genotype; this phenomenon may justify the protective effect of GA/AA genotype in OSA patients. eNOS is one of the three isoforms of NOS enzyme that produces nitric oxide in the presence of BH4 and NADPH^15^. eNOS and NO play an important role in the regulation of endothelial vasodilation, and their functional impairment plays an important role in the development of various diseases^50^, such as cardiovascular diseases^51^, cerebral ischemia^52^, and OSA^53^. Therefore, the expression of eNOS and subsequent endothelial NO release may be affected by gene polymorphism^54^. A study investigated 50 single nucleotide polymorphisms of eNOS in children with OSA and the results suggested that these polymorphisms could contribute to the risk of OSA-induced cardiovascular morbidity^55^. G894T (rs1799983) polymorphism of eNOS is a functional polymorphism which could lead to the sequence change in Glu 298 Asp^56^. Moreover, rs1799983 polymorphism of eNOS gene is associated with reduced activity of NOS and bioavailability of NO. Concurrent presence of CETP B1, NOS3 T, and ANGPTL8 T alleles increases the risk of cardiovascular diseases and type 2 diabetes mellitus^57^.

This study had some limitations. Firstly, the genes selected for investigation had overlap with other diseases such as cardiovascular diseases, diabetes, stroke, and brain ischemia; thus, we only selected patients with no comorbidity. Therefore, it had an advantage and a disadvantage for our study. As an advantage, the genetic assessments were just performed for people who only had OSA; however, as a disadvantage, it was difficult to find patients with no comorbidity, and it resulted in a small sample size. Hence, it is suggested to conduct further studies with larger sample sizes. On the other hand, in order to perform a comparative analysis, it is better to select another group of OSA patients with a concurrent comorbidity. Secondly, we assessed just one polymorphism in each gene, hence the association between other polymorphisms and OSA could be investigated further.

Overall, our study showed that gene polymorphisms in nitric oxide-forming pathway had a reverse association with OSA. rs841 and A930G p22phox polymorphisms had a protective effect in patients with OSA.

## Materials and Methods

### Subjects

This study, as a case-control study, was performed in Baharloo Hospital and Imam Khomeini Hospital, Tehran, Iran. The study protocol was approved by Ethics Committee of Tehran University of Medical Sciences (ethical code: IR.TUMS.VCR.REC.1395.1107). A written informed consent was obtained from all the participants. The experiments were performed in accordance with the American Academy of Sleep Medicine Guidelines^58^.

A total of 94 patients (F19: M75) with OSA and 100 healthy controls (F21: M79) were matched in terms of age and gender. The data on personal characteristics, medical history, and sleep information were obtained through using a questionnaire. All the patients underwent a polysomnography test. Polysomnography was performed overnight, and it monitored many body functions, including skeletal muscle activation (EMG), eye movement (EOG), brain activity (EEG), blood pressure, heart beating, and oxygen saturation. After test analysis, people with 5 ≤ AHI < 15, 15 ≤ AHI < 30 and AHI ≥ 30 were classified into the three groups of patients with mild, moderate, and severe OSA, respectively.

In order to control the costs and consider practical issues, polysomnography was not performed for the controls, and the controls were considered healthy on the basis of data on history that were obtained via answering STOP-BANG and Epworth Sleepiness Scale questionnaires. The cutoff point for STOP-BANG questionnaire was 2^59^, and the cutoff point for Epworth Sleepiness Scale questionnaire was 10^60^.

Exclusion criteria for both case and control groups were the presence of trauma, inflammatory diseases, cardiovascular diseases, brain ischemia, diabetes, chronic pulmonary disorders, asthma, chronic kidney disease, thyroid diseases, smoking history, and drug addiction. Blood samples were collected from the members in the two groups and stored in −20 °C to be used for further examinations.

### DNA extraction and genotyping

DNA was extracted from the whole peripheral blood samples using Geneall DNA extraction kit (Geneall, Seoul, South Korea), in accordance with the manufacturer’s protocol. NADPH Oxidase A930G p22phox was genotyped via Restricted Fragment Length Polymorphism (RFLP) method. The polymerase chain reaction (PCR) forward primer was 5′ GGAAACCACCAAGTGCCTCGGATGG 3′ and Revers primer was 5′ TCTGCACCCTGATGCTACCAAGGAC 3′. PCR was carried out using a volume of 30 ml, under the following condition: an initial denaturation step at 94 °C for 1 min, followed by 31 cycles of 1 min at 94 °C, 1 min at 67 °C, and 1 min at 72 °C; finally, the last elongation step was performed at 72 °C for 2 min. Amplified products were digested using h 3 U of BbvI restriction enzyme for 1 h at 37 °C (New England Biolabs, Beverly, MA, USA). The results of digestion were separated on 3% agarose (Sigma-Aldrich, USA). TaqMan SNP genotyping assays were used for GCH1 rs841 and eNOS rs1799983 genotyping. Following the manufacturer’s protocol, the probes were designed by Applied Biosystems and genotyping were performed on Step-One Plus Real-Time PCR system (Applied Biosystems, Foster City, California, United States).

### Statistical analysis

Statistical analyses were performed in SPSS 25.0 software package for Windows (SPSS Inc, Chicago, IL, USA) and GraphPad Prism 8 (GraphPad Software, San Diego, CA). Chi-square test was preformed to assess deviation from Hardy-Weinberg equilibrium to assess genotypes distribution. The effect of each single-nucleotide polymorphism (SNP) on OSA was investigated using multiple logistic regression analysis adjusted for body mass index in the patients and controls, however, in order to analyze the data obtained from the patient group, multiple logistic regression was preformed after adjusting for age, gender, and body mass index. The strength of the association between the three polymorphisms and OSA was measured via computing ORs at a confidence interval of 95%. Statistical significance was defined as a two-tailed *P* < 0.05.
